# Re-examining the adaptive function of nausea and vomiting in pregnancy

**DOI:** 10.1093/emph/eoae012

**Published:** 2024-07-02

**Authors:** Emily H Emmott

**Affiliations:** UCL Anthropology, University College London, London WC1H 0BW, UK

## Abstract

Nausea and vomiting in pregnancy (NVP) have been proposed to have a prophylactic function. In this review, I re-examine NVP from an evolutionary perspective in light of new research on NVP. First, current evidence suggests that the observed characteristics of NVP does not align well with a prophylactic function. Further, NVP is typically associated with high costs for pregnant women, while moderate-to-severe NVP is associated with increased risks of poorer foetal/birth outcomes. In contrast, mild NVP limited to early pregnancy may associate with improved foetal outcomes—indicating a potential evolutionary benefit. Second, researchers have recently identified growth differentiation factor 15 (GDF15) to cause NVP, with implications that low-levels of pre-conception GDF15 (associated with lower cellular stress/inflammation) may increase risks/symptoms of NVP. If so, NVP in contemporary post-industrialized populations may be more severe due to environmental mismatch, and the current symptomology of NVP in such populations should not be viewed as a typical experience of pregnancy.

## INTRODUCTION

Nausea and vomiting in pregnancy (NVP) is a common condition estimated to impact approximately 70% of pregnant women (according to data primarily from post-industrialized populations) [1]. Until relatively recently, even into the 21st century, NVP was often assumed to be a psychosomatic symptom where psychological trauma relating to the pregnancy manifested as physical symptoms of vomiting and nausea [2]. This flawed assumption stifled research advancements, with very little scientific attention paid towards understanding NVP. Over the past decade, however, we have seen more research on the wide-ranging impact of NVP on women and their offspring [3]. Recently, researchers have finally successfully identified one of the physiological mechanisms underlying NVP, where growth differentiation factor 15 (GDF15) has been causally linked to NVP prevalence and severity [4, 5]. For evolutionary scholars, these new advancements in the field are highly exciting: NVP is not typically observed among non-human animals [6], which makes us question why. With an improved understanding of the fitness consequences and mechanisms behind NVP, we are in better position to consider why NVP exists in humans. In this review, I revisit existing evolutionary theories on NVP in light of recent research findings.

### What is NVP?

While NVP is sometimes incorrectly referred-to as ‘morning sickness’, most women experience NVP at any time of day [1]. For the majority of pregnancies, NVP symptoms begin to ease after the first trimester, but current estimates suggest one in four women will continue to experience NVP into their third trimester [1]. NVP is typically viewed as a ‘normal’ side-effect of pregnancy, but more severe cases leading to dehydration, weight loss, and ketosis is viewed as pathological and may be diagnosed as *hyperemesis gravidarum*. Due to the under-diagnosis and under-recording of *hyperemesis gravidarum*, its prevalence is challenging to ascertain—but recent studies suggest *hyperemesis gravidarum* may present in 0.3–10.8% of pregnant women [3].

NVP, even in milder forms, is associated with a significant reduction in quality of life and wellbeing [7, 8] and increased risks of antenatal and postpartum depression [9–12]. In the USA alone, NVP is estimated to lead to an average of 23 days lost from work, with a total economic burden of $1.7 billion [13]. While acknowledging these negative experiences and consequences of NVP, evolutionary scholars have previously argued that NVP (excluding *hyperemesis gravidarum*) may be an adaptation that ultimately leads to greater maternal reproductive success. The prophylactic function hypothesis suggests that NVP may protect the foetus from potentially harmful toxins and pathogens, which may be particularly important during the first trimester where there are greater foetal vulnerabilities combined with systemic maternal immunosuppression and higher risks of spontaneous abortion [6, 14–18]. The maternal-and-embryo-protection hypothesis additionally proposes direct benefits for mothers, whereby NVP mitigates against the increased risks of infectious diseases caused by the pregnancy-related immunosuppression [6, 19]. These hypothesized inclusive fitness benefits may outweigh the costs of NVP, especially in the first trimester where maternal energy requirements remain the same as pre-conception [20].

In support, NVP is most commonly experienced in the first trimester which correlates with the period of greatest foetal and maternal vulnerability [6]. Further, food aversions have been described to be targeted towards foodstuff with greater infection/toxicity risks, such as meat, alcohol, and caffeine [6]. A comparative study of country-level dietary characteristics and NVP prevalence found that higher population-level consumption of animal products, alcohol, and stimulants (among other things) are associated with increased NVP prevalence [21]. This suggests mothers may be more likely to experience NVP in populations contexts where there is higher exposure to ‘risky’ and ‘toxic’ foods, supporting the hypothesis that NVP may indeed have a prophylactic function. In addition, some studies have found that NVP is associated with lower risks of miscarriage [22, 23], while analysis of cohort studies from Norway and Japan both found NVP to be associated with better birth outcomes, including full-term birth and higher birthweight [24, 25], suggesting NVP may translate to better foetal quality overall.

### Is NVP adaptive? Current evidence

While the aforementioned studies seem to support NVP as an adaptation, careful consideration of the causal pathways is required. For example, rather than NVP directly causing lower miscarriage risk, it may be that NVP is primarily experienced by those with viable pregnancies [26] – and the association between NVP and lower miscarriage risk may be an analytical by-product of survivorship bias. It is therefore crucial to carefully map-out the costs and benefits associated with NVP, as well as consider the mechanisms underpinning these associations. Below, I explore current evidence on any benefits associated with NVP and consider the implications for functional explanations of NVP.

#### Does NVP function to reduce exposure to infections/toxins?

Both the prophylactic function hypothesis and the maternal-and-embryo-protection hypothesis propose that NVP-related nutritional restriction and food aversions reduce the risks of infection and toxin exposure, consequently reducing miscarriage risk. A key component of these adaptive hypotheses is targeted food aversions, particularly around animal products which have increased risks of infections (e.g. toxoplasma infections from meat, brucellosis infections from unpasteurized dairy consumption) [6]. While detailed studies of maternal diet and NVP are scarce, a Finnish study found that NVP was associated with reduced meat consumption in line with this hypothesis [27]. However, women with NVP in this study also reduced their vegetable intake [27], meaning it is not clear whether food aversions were specifically targeted towards ‘high-risk’ foods.

In fact, several studies show that the diets of women who experience NVP are typically less optimal for maternal–foetal health compared to those who do not experience NVP: in one study conducted in China, women with NVP had comparatively lower intake of energy overall, with lower consumption of protein, fat, vitamin A, iron, potassium, zinc to name a few [28], indicating they were eating less food in general. In a study conducted in Norway, women experiencing NVP consumed proportionally higher levels of carbohydrates which was largely driven by greater intake of sugar-containing soft drinks [26], while in a UK birth cohort study, women who had severe NVP tended to reduce their food consumption while increasing intake of white bread and soft drinks [29]—meaning NVP was generally associated with a poorer diet. Although it is important to note that there is great variation in the impact of NVP on maternal diet [29], these findings raise questions about whether pregnant women are reducing consumption of solid food as a coping mechanism to NVP [26], rather than avoiding specific ‘risky’ foods which may contain pathogens or toxins. If food-avoidance is general rather than targeted, careful consideration must be made on how these dietary changes in pregnancy would be beneficial for maternal-foetal outcomes, as it may increase the risk of nutritional deficiencies [26–28]. Even in the 1st trimester, micronutrient deficiencies and poorer diets are associated with poorer birth outcomes [30, 31]. While rare, cases of maternal brain damage (Wernicke’s encephalopathy) due to acute thiamine deficiency caused by hyperemesis gravidarum have been reported [32]. Further, it is important to note that most ‘risky infections’ during pregnancy are not foodborne (e.g. malaria, dengue fever, flu) [33]. Given the importance of adequate nutritional status to resist/fight infections [34], it is not immediately clear how general food aversions will lead to an inclusive fitness benefit.

Further, if one assumes a protective effect of NVP via avoidance of food-related infection/toxicity, we may predict a greater prevalence of NVP among women with higher risks of infection (such as those experiencing immunosenescence), toxin exposure (such as smokers), and miscarriage overall. However, NVP risk is more common among younger women [26], even though older women are more likely to benefit from the prophylactic function of NVP as they hold greater risks of miscarriage and comparative immunosenescence [35, 36]. Other studies show that pre-conception alcohol consumption and smoking are broadly associated with *lower* levels of NVP during pregnancy [26, 28, 37, 38] (although once NVP is emerges it may lead to reduced alcohol consumption/smoking [37]). Again, if NVP has a prophylactic function, we may expect NVP prevalence to be higher among women who have greater exposure to toxins, particularly in regard to smoking due to its robust association with immunosuppression, increased vulnerabilities to infection, and miscarriage [39, 40]. Of course, one could argue that Tabacco exposure is relatively novel in Europe and Asia [41], therefore evolved prophylactic mechanisms of NVP may not adequately compensate for the increased risks associated with smoking. Nonetheless, current studies suggest that, at the individual level, women who are more likely to benefit from NVP are typically not more likely to experience NVP.

Taken together, current evidence around how NVP presents among pregnant women does not align well with the evolutionary hypothesis that NVP has a prophylactic function. In contrast, there is robust evidence that describes the broader costs of NVP in pregnant women beyond food aversion/nutritional restriction: even in mild cases, NVP is frequently associated with the inability to carry out day-to-day tasks, including work/production activities, looking after existing children, and maintaining social connections [42, 43]—which are all potentially highly costly for maternal inclusive fitness. While social withdrawal in early pregnancy could be beneficial in reducing infection from contagious diseases (such as flu), whether or not the observed costs are outweighed by potential benefits are likely to be context-dependent. In contemporary post-industrialized populations with lower risks from infectious diseases, combined with a dual-burden of maternal production and caregiving (where many mothers are simultaneously in paid employment while being primary caregivers), the costs of NVP may be particularly high with little benefit. Given the symptoms, it is not surprising that NVP is strongly associated with reduced quality of life and antenatal/postpartum depression, which in itself is associated with a myriad of poorer offspring outcomes, including low birth weight, pre-term birth, and poorer mental health associated with conduct problems and antisocial behaviour [44–46]. As it stands, there is very limited evidence of any direct benefits mothers gain via NVP, bringing into question what exactly the benefits associated with NVP could be (if there is one).

#### Does NVP benefit foetal quality?

Regardless of whether NVP evolved to protect pregnant women and/or foetuses against infections/toxins in early pregnancy, the fact that NVP is observed across human populations, and how NVP is rarely observed in non-human animals, has been presented as an argument that NVP must have been under positive selection in humans [6]. Studies identifying associations between NVP and reduced miscarriage risk [22, 23] further supports the proposition that NVP is adaptive and brings benefits to foetal quality [6]. However, the association between NVP and foetal outcomes are in fact mixed, with studies finding NVP to associate with increased, decreased, and no differences in the risks of low birthweight and pre-term births [47].

The lack of consistency in the association between NVP and foetal quality may stem from inconsistencies in how NVP is defined. The intensity, duration, and the symptomology of NVP is highly variable within and between populations. In fact, studies which focus on more severe forms of NVP (including, but not limited to, *hyperemesis gravidarum*) consistently find a general association between NVP and preterm birth, low birthweight and foetal growth restriction, as well as some evidence of rarer outcomes such as neurodevelopmental delay, vitamin K deficient embryopathy, autism, respiratory issues, cardiovascular issues, and cancer [48–53]. These poor birth outcomes may lead to longer-term detrimental impacts on offspring, with severe NVP associated with increased risk of psychological issues including ADHD, depression, and socio-emotional difficulties among children in US and Danish cohorts [54]. MRI brain scans of the US cohort identified lower cortical volume among children whose mothers experienced severe NVP, which mediated the relationship between maternal NVP and offspring cognitive/psychiatric risks [54]. A recent prospective cohort study from England with comparatively robust methods found that vomiting (but not nausea) in pregnancy, even with mild symptoms, was associated with lower birth weight [55]. Similarly, a study from China found that vomiting in the first trimester was associated with preterm birth [56]. If vomiting is a particular risk factor for poorer foetal/birth outcomes, this further explains study inconsistencies, as women who experience nausea without vomiting can also be classed as having NVP.

Beyond the severity and symptomology of NVP, there is some evidence that the timing and duration of NVP also matters. In a recent analysis of the US National Birth Defects Prevention Study, NVP in the first trimester was associated with a reduced risk of small-for-gestation-age birth, in line with earlier studies which found a general positive association between NVP and better birth outcomes [57]. However, in the same study, NVP lasting beyond early pregnancy into the second and third trimester was associated with an increased risk of preterm birth [57]. Similarly, in an analysis of the Swedish Medical Birth Register, women who experienced in-patient treatment for HG in the first trimester only had a slight increased risk of pre-eclampsia compared to baseline, while women who experienced in-patient treatment for HG in the second trimester experienced significant increased risks of pre-term pre-eclampsia, placental abruption and small-for-gestational-age birth [58]. The difference in outcomes between NVP in early vs later pregnancy may relate to how nutritional restriction specifically in early pregnancy may be associated with increased placental growth to compensate for reduced maternal nutrition [16]. This was demonstrated in the Dutch Hunger Famine, where women who experienced the famine in the first trimester experienced increased placental growth without impact on birthweight [59]. Similar patterns have been experimentally demonstrated in sheep and mice, although findings are not consistent across other mammals [60]. As placenta size broadly predicts foetal growth in humans (excluding notably large/thick placentas which predicts poor foetal/birth outcomes) [61], NVP may therefore be an adaptation to encourage foetal development [16]—although it is important to note that mild NVP is not necessarily associated with reduced food intake in early pregnancy [29]. Nonetheless, all these studies show how prolonged or severe NVP may have a different cost-benefit profile compared to milder NVP in early pregnancy, explaining the frequent inconsistencies in current research.

Overall, current evidence provides robust evidence that moderate-to-severe NVP, which is very common, is associated with poor foetal/birth outcomes. For milder cases, however, the evidence is mixed. There is some evidence to suggest that milder NVP, particularly when limited to the 1st trimester, possibly without vomiting, is associated with better birth outcomes. This association could potentially be explained by the association between nutritional restriction in early pregnancy and increased placental growth, suggesting that nausea-induced early food aversion could have evolved to encourage foetal development, although more research is needed. Regardless, current literature highlights that the costs and benefits of NVP may be highly variable depending on the characteristics of NVP, and these variations must be considered when reflecting on NVP from an evolutionary perspective.

As it stands, current correlational evidence surrounding NVP and maternal–offspring outcomes do not present strong evidence of adaptive benefits for NVP more broadly. Over the last decade, the potential costs of NVP for pregnant women and their offspring have been more clearly identified, and any benefits seem to be limited to mild cases of NVP in early pregnancy. (As mentioned, current estimates suggest 1 in 4 women will continue to experience NVP into their third trimester [1].) This is not to say that NVP is maladaptive; indeed, any claims of NVP being maladaptive requires equal scrutiny, particularly given its high prevalence across populations. Rather, the point here is that the relationship between NVP and fitness benefits are not clear from the available evidence. Within this ambiguity of evidence, an understanding of the underlying physiological mechanisms behind NVP may be particularly useful, allowing us to critically reflect on the plausibility of NVP as an adaptation (or maladaptation).

### Physiological mechanisms behind NVP

The aetiology of NVP has been unclear until relatively recently. Several previous studies indicated positive associations between NVP and human chronic gonadotrophin (hCG) [62], a ‘pregnancy hormone’ central to promoting maternal immunotolerance of the foetus [63]. However, the exact pathways between NVP and hCG have remained unclear, while several studies failed to find a link between NVP and hCG [62, 64].

More recently, studies have indicated a causal link between a different hormone, growth differentiation factor 15 (GDF15), and NVP. GDF15 is a pleiotropic hormone associated with multiple physiological processes including appetite regulation, metabolic regulation, and anti-inflammatory effects [5]. Considered a stress-responsive cytokine, elevated GDF15 expression is associated with increased cell-stress and inflammation such as after exercise, infection and injury [5, 65]. GDF15 expression is particularly high in the placenta; and while its function is not yet fully clear, maternal-foetal immunotolerance has been suggested [66], and low GDF15 has been associated with increased miscarriage risk (although the causal direction is still unclear) [66, 67]. Previous studies had linked GDF15 with loss of appetite and cachexia in mice and patients with cancer [5, 62, 68], indicating a relationship with NVP-relevant symptoms. Several observational studies then found that higher concentrations of circulating GDF15 is associated with more severe cases of NVP [4, 62], complementing the findings that the gene loci for GDF15 is associate with *hyperemesis gravidarum* [48]. In 2023, Fejzo *et al*. directly linked circulating GDF15 of foetal/placental origin to NVP, and demonstrated that maternal sensitivity to GDF15 moderates this relationship, explaining variations in NVP symptoms [4].

As expected, maternal sensitivity to GDF15 is influenced by both genetic, physiological, and environmental factors. For example, the rare coding variant, C211G, for GDF15 has been associated with presence of the most severe form of NVP [69]. Women with beta-thalassaemia who have chronically high levels of GDF15, who consequently develop reduced sensitivity to GDF15, report lower levels of NVP despite their comparatively higher levels of GDF15 in pregnancy [4]. Importantly, this indicates that *pre-conception GDF15 levels are likely to directly influence the risk of NVP*: prolonged and high levels of circulating GDF15 pre-pregnancy is likely to reduce sensitivity to GDF15, leading to greater tolerance of placental GDF15 in pregnancy, and consequently lowering the risk of NVP. This may explain why smoking pre-pregnancy is associated with low NVP, as smoking has been associated with cellular stress and increased levels of circulating GDF15 [70]—which may cause greater ‘GDF15 tolerance’ and protection against NVP. GDF15 also increases with age, presumably due to increased cellular stress, and has been used as a biomarker associated with ageing [70]. This, again, explains the association between older women and reduced risk of NVP—where elevated pre-conception GDF15 levels reduce maternal sensitivity to placental GDF15.

### Is there a mismatch? Increased severity of nausea and vomiting during pregnancy

The recently revealed mechanism of GDF15 in NVP is particularly interesting, as it brings insight to how contemporary post-industrial environments may impact NVP severity. As noted above, GDF15 expression is associated with increased cell-stress and inflammation [5, 65], and low levels of pre-conception GDF15 may make women more susceptible to NVP (as they may be more sensitive to placental GDF15 [4]). In post-industrialized populations with lower exposure to pathogens and lower engagement with intense physical activity [71–74], women may be expressing comparatively lower levels of preconception GDF15 compared to non-industrialized settings. If so, the typical NVP symptoms experienced across contemporary populations may be more severe than what was the norm in our recent evolutionary history. Moderate to severe forms of NVP (experienced by many pregnant women), as well as *hyperemesis gravidarum* which is already acknowledged to be pathological, may be a direct consequence of environmental mismatch.

If environmental mismatch exists, the overall implication is that ‘functional’ NVP may be limited to particularly mild forms of NVP. Indeed, as reviewed above, there is some evidence that milder forms of NVP limited to early pregnancy may encourage placental growth and improve birth outcomes [16, 57] – and it is possible that mild NVP was positively selected in humans due to this benefit. However, to date, research on NVP from an evolutionary perspective has typically treated NVP (excluding *hyperemesis gravidarum*) as a homogenous category without careful consideration of the variations in severity, symptoms, and duration. Further research is therefore necessary to explore and test this mismatch hypotheses. For example, it would be interesting to investigate variations in NVP severity and duration across different population and sub-population contexts, testing the prediction that NVP severity and duration is more likely to be milder and shorter in non-industrialized populations.

## Conclusion

An evolutionary approach to understanding NVP is important for understanding what is ‘normal’ for pregnancy. This may have direct implications on how we view and treat NVP symptoms. To date, the high prevalence of NVP observed in contemporary populations have led to the view that nausea and/or vomiting is a normal part of pregnancy. Based on this assumption, various adaptive hypotheses have been proposed. However, recent studies evidence clear costs of NVP for the mother with no apparent direct fitness benefits. Further, there is no strong evidence to suggest NVP is broadly beneficial for the foetus/offspring, although mild NVP in early pregnancy may promote foetal growth. A consideration of the recently uncovered causal pathways behind NVP, namely foetal GDF15 production, suggests symptoms of NVP in contemporary post-industrialized populations may be more severe due to environmental mismatch. If so, the current symptomology of NVP in such populations should not necessarily viewed as a typical experience of pregnancy.

Further research is clearly needed to test the mismatch hypothesis with careful consideration of variations in NVP. This is particularly important, as improving understanding NVP as mismatch may lead to implications for practice and patient experience. In Western populations, there is a long and complex history of dismissing NVP symptoms which persists today: NVP is often not recorded in medical records due to the assumption that it is ‘normal’, meaning we have a poor understanding of NVP symptomology and prevalence [1]. Women are still routinely not listened-to, with healthcare practitioners frequently failing to offer adequate treatment [75, 76]. Grounded in evolutionary theory, shifting the understanding of moderate to severe NVP as potentially harmful for maternal wellbeing and foetal outcomes may facilitate better monitoring, support, and active treatment.
